# The Impact of Memory Stereotype Threat on Memory and Memory Self-Efficacy in Older Adults

**DOI:** 10.1093/geroni/igaa057.1047

**Published:** 2020-12-16

**Authors:** Lauren Fredriksen, Renee’ Zucchero, Brock Partlow, Ruth Infante, Janie Taylor, Haley Washburn

**Affiliations:** Xavier University, Cincinnati, Ohio, United States

## Abstract

This study examined the impact of memory stereotype threat on memory duration (e.g., short-term and long-term) and modality (e.g., verbal and non-verbal), and memory self-efficacy in older adults who live independently (Mage = 77 years). Participants (N= 66) were randomly assigned to a group that received either neutral instructions or memory stereotype threat inducing instructions. All participants completed the California Verbal Memory Test-Second Edition (CVLT-2), the Rey Complex Figure Test (RCFT), a memory self-efficacy measure, and a demographics survey. An independent samples t-test indicated participants in the stereotype threat group reported significantly lower memory self-efficacy than participants in the neutral group. The main effect of the within-subjects factor of a 2x2 mixed analysis of variance (ANOVA) indicated that participants performed significantly better on short-term non-verbal memory than long-term non-verbal memory. There was no significant difference between the neutral and stereotype threat groups in memory modality or duration. These results may indicate that the instructions used to induce memory stereotype threat were not phrased strongly enough to elicit poorer performance on the CVLT-2 and RCFT in the memory stereotype threat group. Additionally, participants reported having a high level of education (i.e., a master’s degree was the modal educational level), which may have served as a buffer for memory stereotype threat. The findings call for future research to explore the impact of level of education on memory self-efficacy in older adults. Also, future research may focus on the impact of the strength of memory stereotype threat on memory performance.

